# Cardiovascular events and mortality in a population-based cohort initially diagnosed with ductal carcinoma in situ

**DOI:** 10.1186/s12885-021-08494-0

**Published:** 2021-06-26

**Authors:** Tae-Kyung Yoo, Sang Hyun Park, Kyung Do Han, Byung Joo Chae

**Affiliations:** 1 Department of Surgery, Seoul St. Mary’s Hospital, College of Medicine, The Catholic University of Korea, Seoul, 06591 Republic of Korea; 2 Cancer Research Institute, College of Medicine, The Catholic University of Korea, Seoul, 06591 Republic of Korea; 3 Department of Medical Statistics, College of Medicine, The Catholic University of Korea, Seoul, 06591 Republic of Korea; 4 Department of Statistics and Actuarial Science, Soongsil University, Seoul, 06978 Republic of Korea; 5 Division of Breast Surgery, Department of Surgery, Samsung Medical Center, Sungkyunkwan University School of Medicine, 81 Irwon-Ro, Kangnam-Gu, Seoul, 06531 South Korea

**Keywords:** Ductal carcinoma in situ, Mortality, Myocardial infarct, Stroke, Cardiovascular event, Population-based cohort

## Abstract

**Background:**

Ductal carcinoma in situ (DCIS) patients are usually diagnosed through cancer screening programs, suggesting a healthy user effect. In this population-based cohort, we assessed the risk of cardiovascular events and mortality in DCIS patients.

**Methods:**

Using the Korean National Health Insurance Service database, 13,740 women, who were initially diagnosed with DCIS between 2007 and 2013, were analyzed. A control group was matched according to age and the year of diagnosis at a 3:1 ratio (*n* = 41,220). Follow-up was performed until 2016. Subgroup analysis was performed according to the subsequent diagnosis of invasive breast cancer within 1 year: pure DCIS and DCIS+Invasive group.

**Results:**

DCIS patients were more likely to have underlying diseases, higher incomes, and to live in urban districts compared to the control group. Women diagnosed of DCIS had lower myocardial infarct risk (hazard ratio [HR] 0.64; 95% confidence interval [CI] 0.46–0.90) and lower stroke risk (HR 0.77; 95% CI 0.60–0.98) compared to the control group. This trend of lower risk was sustained after adjusting for age, income, residence and comorbidities. The mortality rate was similar between the control group and pure DCIS patients but was higher in the DCIS+Invasive group (HR 1.63; 95% CI 1.34–1.98). However, after adjusting for age, income, residence and comorbidities, mortality did not differ between the control group and DCIS+Invasive group (HR 0.99; 95% CI 0.78–1.24).

**Conclusions:**

DCIS patients were at lower risk for MI and stroke compared to a control group despite a higher rate of comorbidities, which may reflect changes in health behaviour. The importance of managing pre-existing comorbidities along with DCIS treatment should be emphasized.

**Supplementary Information:**

The online version contains supplementary material available at 10.1186/s12885-021-08494-0.

## Background

The incidence of ductal carcinoma in situ (DCIS) has increased worldwide, mainly related to the introduction of breast screening programs [1–5]. This phenomenon has also been identified in Korea after breast cancer screening started in 2002. In Korea, breast cancer screening is provided by the National Health Insurance System, via mammography to all women every 2 years starting from the age of 40 years. Screening rates steadily increased from 9.4% in 2002 to 59.7% in 2013 and the proportions of DCIS and early breast cancer increased significantly during this period [6, 7].

Women who adhere to mammography screening are suggested to have a ‘healthy user’ effect [8]. As most DCIS patients are detected by screening programs, women diagnosed with DCIS can also present with the healthy user effect. This means that DCIS patients might generally be in better health, be more health-conscious, have fewer comorbidities, and have a higher socioeconomic status. Two large population-based studies demonstrated lower mortality in DCIS patients, possibly reflecting the healthy user effect [8, 9]. Ernster et al. used data from the Surveillance, Epidemiology, and End Results (SEER) program and reported a significantly lower 10-year standardized mortality ratio (SMR) of 0.8 (95% confidence interval [CI], 0.7–0.8) [9]. Similarly, Elshof et al. used the Netherlands Cancer Registry data, which also revealed lower mortality in DCIS patients compared to the general population (SMR 0.92; 95% CI, 0.87–0.97) [8].

Among women diagnosed with DCIS, breast cancer-related mortality is very low, with recent data from the SEER registry demonstrating a 3.2% breast cancer-related mortality after 20 years of follow-up [10]. In comparison, the cumulative risk at 20 years of follow up for death due to cardiovascular disease was 13.2%, being the leading cause of mortality among DCIS patients. However, when comparing cause-specific mortality with the general population, CVD-related mortality in DCIS patients present with similar or decreased CVD-related mortality [8, 9, 11, 12]. In consideration of the healthy user effect DCIS patients, a low CVD risk could be expected, despite of radiation exposure of the heart, which has relatively low dose [8–12]. Women diagnosed with DCIS are generally in good health and typically have no symptoms related to their diagnosis. Many patients overestimate their risk perceptions after a DCIS diagnosis, provoking unnecessary anxiety and psychological distress [13, 14]. Accurate information on the risks of DCIS should be provided to patients and healthcare providers to minimize these inaccurate perceptions.

Several population-based studies have investigated cause-specific mortality among DCIS patients, but few have assessed comorbidities and cardiovascular events related to these comorbidities. In this population-based cohort, we assessed the likelihood of cardiovascular events and mortality in DCIS patients, compared with a healthy control group after adjusting for potential confounders, such as comorbidities and socioeconomic status.

## Methods

### Korean National Health Insurance System database

The National Health Insurance System (NHIS) is a nonprofit single-payer organization run by the Korean government. Almost all Korean citizens (97.2%, ~ 50 million) are covered by the NHIS, with the remaining 3% with low income covered by the Medical Aid Program. The NHIS database contains extensive health information datasets regarding demographics, medical treatment, procedures, disease diagnoses according to the International Classification of Diseases, 10th Revision (ICD-10) codes, and health examinations.

### Study cohort

**Fig. 1:**
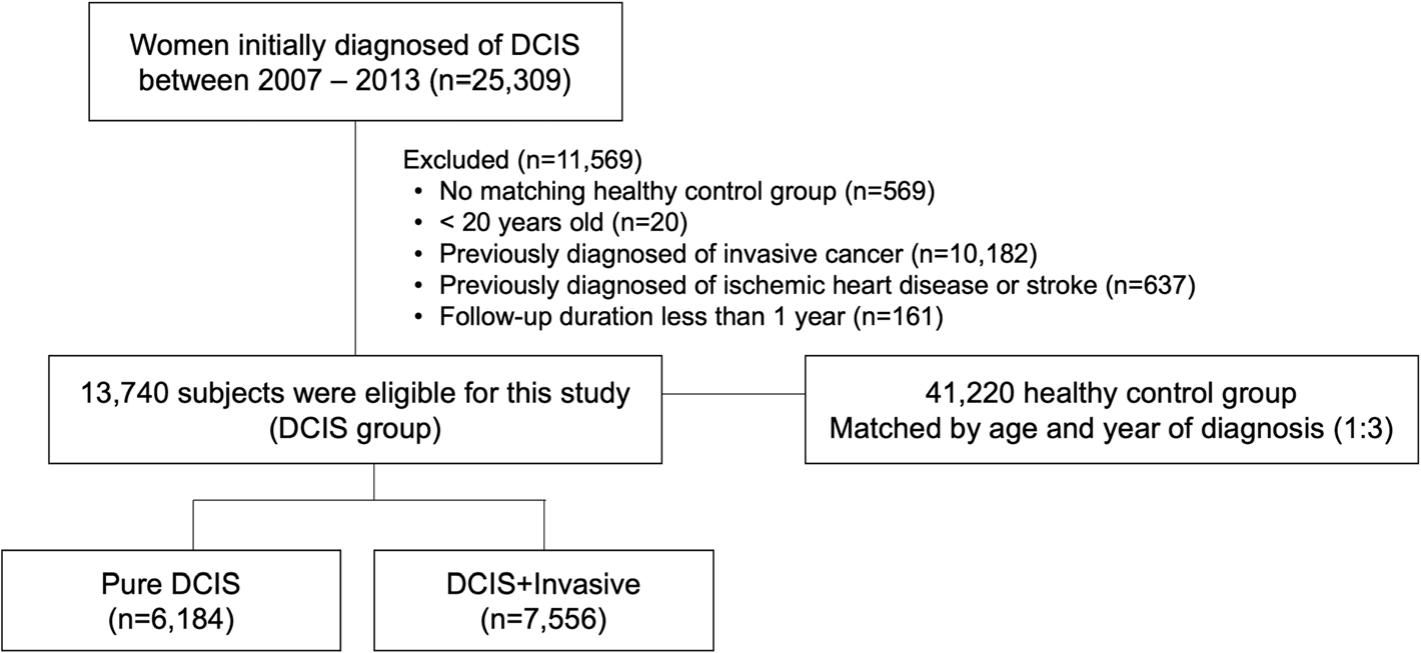
Flowchart of the study population

This study used the NHIS claims dataset from the period January 2002 to December 2016. A 5-year washout period was applied from 2002 to 2006 to exclude previous cancer or cardiovascular disease (CVD) diagnoses. Women who were diagnosed with DCIS between 2007 and 2013 were included in this study (*n* = 25,309; Fig. 1). Of these, patients were excluded if they met any of the following criteria: patients without any matching control group, patients < 20 years old, patients with a history of any invasive cancer, ischemic heart disease, or stroke, and patients with a follow-up duration < 1 year. In total, 13,740 women were included in this study as the case group, indicated as the DCIS group. A comparison cohort of women without breast cancer was matched to the DCIS group, after applying the same exclusion criteria. The control group was matched to the DCIS group according to age and year of diagnosis at a 3:1 ratio (*n* = 41,220). The DCIS group was divided into two subgroups according to the diagnosis of subsequent invasive breast cancer within 1 year: the pure DCIS group and the DCIS+Invasive group.

### Assessment and definitions

Comorbidities were defined based on the ICD-10 codes and the use of related medications (Additional file 1). Hypertension was defined by the ICD-10 codes I10–13 and I15 with a prescription of related medications. Type 2 diabetes mellitus (DM) was defined by the ICD-10 code of E11–14 with a prescription of related medications. Dyslipidemia was defined by the ICD-10 code of E78 with a prescription of related medications. The components of the Charlson Comorbidity Index (CCI) were defined from ICD-10 codes from outpatient and inpatient claims [15, 16]. These were all identified from prescriptions and diagnosis codes claimed in the same year of DCIS diagnosis. Household income was determined by the health insurance payment rate, which is calculated from yearly income.

The two outcomes for CVD were myocardial infarct (MI) and stroke, which were identified 1 year after the DCIS diagnosis. The diagnosis of MI was identified based on ICD-10 codes (I21, I22) during hospitalization. The stroke diagnosis was defined by ICD-10 codes (I63, I64) during hospitalization along with claims for brain imaging studies (brain computed tomography or magnetic resonance imaging). Mortality data were obtained by merging the NHIS claims data and the national mortality data from the Korean National Statistical Office. Mortality data was also identified after 1 year from DCIS diagnosis.

This study was approved by the NHIS inquiry commission, and all data were provided after de-identification. This study was also approved by the Institutional Review Board of Seoul St. Mary’s Hospital (IRB no. KC18ZESI0157) and the need to obtain informed consent was waived.

### Statistical analysis

The characteristics of the DCIS and control groups were compared using the χ^2^ test and one-way analysis of variance. A Cox proportional hazards regression analysis was performed to evaluate the associations between DCIS and cardiovascular events or mortality. Model 1 was the crude model, model 2 was adjusted for age, and model 3 was adjusted for age, income, DM, hypertension, and dyslipidemia. Kaplan–Meier curves were prepared to show the cumulative incidence of MI, stroke, and mortality, and a log-rank test was performed to examine the association between DCIS and the risk of cardiovascular events or mortality. A *p*-value < 0.05 was considered significant. All analyses were performed with SAS (version 9.4; SAS Institute, Cary, NC, USA).

## Results

### Patient characteristics

In total, 13,740 women were diagnosed with DCIS between 2007 and 2013. Among them, 6184 (45.0%) women had only DCIS (pure DCIS group) and 7556 (55.0%) women had a subsequent diagnosis of invasive breast cancer within 1 year of the DCIS diagnosis (DCIS+Invasive group). The median age at diagnosis was 47.7 years. The patient characteristics are compared to the control group in Table 1. Patients with DCIS were more likely to have underlying diseases, such as hypertension, DM, and dyslipidemia, and to have a higher CCI score compared to those in the control group. The DCIS group also had a higher household income and was more likely to live in an urban area compared to the control group.

**Table 1:** Baseline Characteristics of DCIS and Control Groups

		DCIS group *N* = 13,740 N (%)	Control group *N* = 41,220 N (%)	*P* value
Age	Mean (SD)	47.68 (9.68)	47.68 (9.68)	1.000
Hypertension		2179 (15.86)	5907 (14.33)	< 0.001
DM		692 (5.04)	1860 (4.51)	0.0115
Dyslipidemia		1479 (10.76)	2938 (7.13)	< 0.0001
CCI	Mean (SD)	2.64 (1.42)	1.73 (1.17)	< 0.0001
Income by quartile	Q1	3431 (24.97)	12,606 (30.58)	< 0.0001
	Q2	2738 (19.93)	9248 (22.44)	
	Q3	3163 (23.02)	9351 (22.69)	
	Q4	4408 (32.08)	10,015 (24.30)	
Urban area residence		7377 (53.69)	19,514 (47.34)	< 0.0001

*DM* Type 2 diabetes mellitus, *CCI* Charlson Comorbidity Index, *MI* myocardial infarct, *SD* standard deviation

### Myocardial infarct, stroke, and mortality risk

**Fig. 2:**
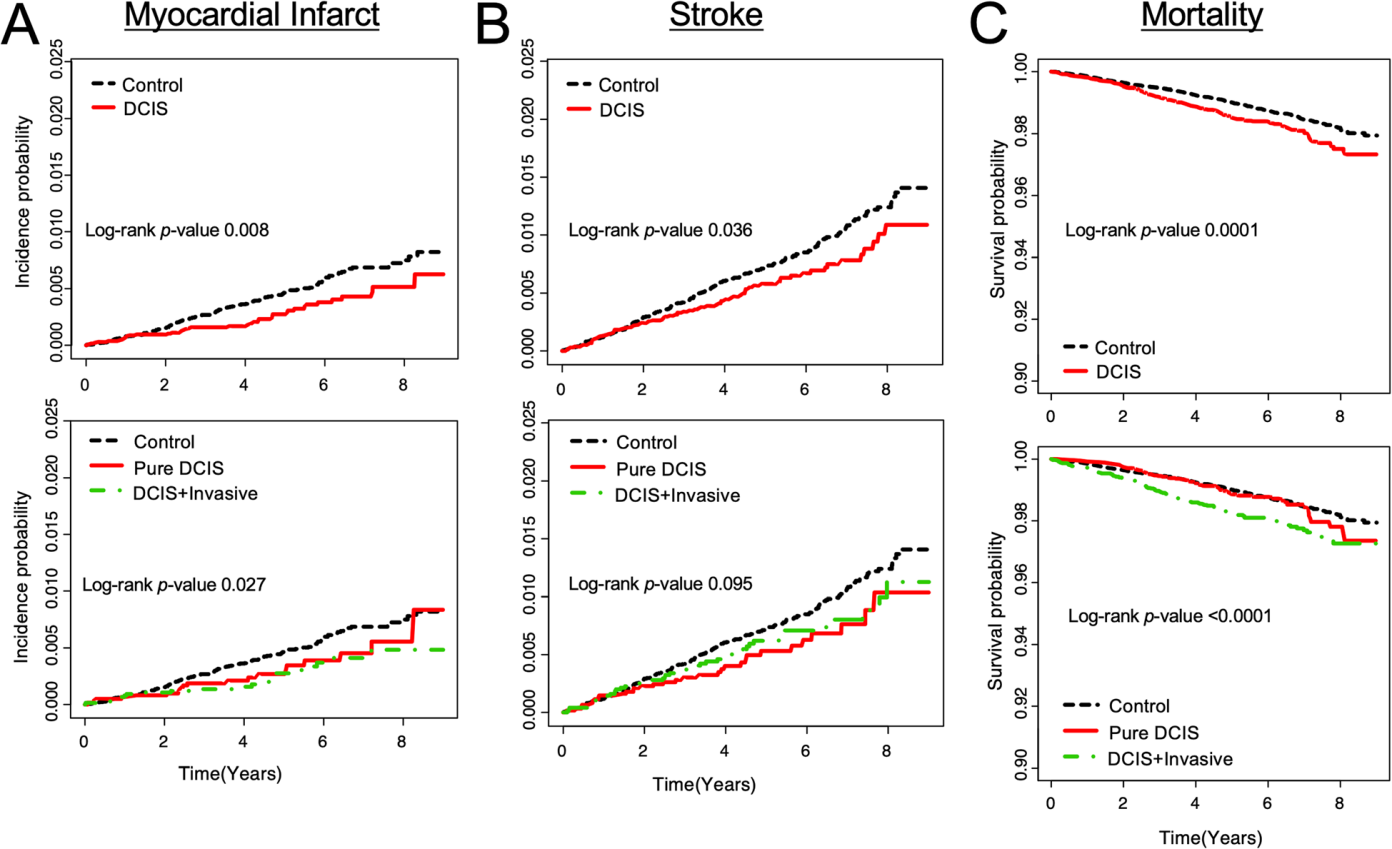
Kaplan-Meier estimates of the cumulative incidence of myocardial infarct (**A**), stroke (**B**), and mortality (**C**) in women initially diagnosed with ductal carcinoma in situ (DCIS) compared to a healthy control group. A subgroup analysis according to subsequent invasive breast cancer is also presented

The median follow-up duration was 5.36 years (Q1–Q3, 3.26–6.41). During the follow-up, 234 women were diagnosed with MI, 388 women were diagnosed with a stroke, and 625 women died. The DCIS group had a significantly lower MI risk (hazard ratio [HR] 0.64; 95% CI, 0.46–0.90) and a lower stroke risk (HR 0.77; 95% CI, 0.60–0.98) than the control group (Table 2, Fig. 2). The lower MI and stroke risks were sustained after adjusting for age (model 2), after adjusting for age, income, DM, hypertension, and dyslipidemia (model 3) or after adjusting for age, income, residence, DM, hypertension, dyslipidemia and CCI score (model 4). The mortality rate was higher in the DCIS group than the control group (HR 1.340; 95% CI, 1.18–1.65). However, after adjusting for age, income, residence and chronic diseases (model 4), mortality rate did not differ between the control group and DCIS group (HR 0.97; 95% CI, 0.80–1.17).

**Table 2:** Risk of myocardial infarct, stroke and mortality in DCIS group compared to the control group

		Total N	Events (n)	Follow-up Duration (Person-Year)	Incidence rate per 1000	Model 1^a^ HR (95% CI)	Model 2^b^ HR (95% CI)	Model 3^c^ HR (95% CI)	Model 4^d^ HR (95% CI)
Myocardial Infarct	Control group	41,220	193	205,501	0.939	1	1	1	1
	DCIS group	13,740	41	68,411	0.599	0.64 (0.46, 0.90)	0.64 (0.46, 0.89)	0.63 (0.45, 0.89)	0.51 (0.36, 0.74)
	▪ Pure DCIS	6184	20	30,104	0.664	0.71 (0.45, 1.13)	0.69 (0.44, 1.10)	0.69 (0.43, 1.09)	0.63 (0.40, 1.01)
	▪ DCIS+Invasive	7556	21	38,307	0.548	0.58 (0.37, 0.91)	0.59 (0.38, 0.93)	0.59 (0.37, 0.92)	0.42 (0.26, 0.68)
Stroke	Control group	41,220	309	205,246	1.506	1	1	1	1
	DCIS group	13,740	79	68,315	1.156	0.77 (0.60, 0.98)	0.77 (0.60, 0.98)	0.76 (0.59, 0.97)	0.60 (0.46, 0.79)
	▪ Pure DCIS	6184	32	30,076	1.064	0.71 (0.50, 1.02)	0.69 (0.48, 0.99)	0.68 (0.47, 0.98)	0.63 (0.44, 0.91)
	▪ DCIS+Invasive	7556	47	38,239	1.229	0.82 (0.60, 1.11)	0.83 (0.61, 1.13)	0.82 (0.60, 1.12)	0.58 (0.41, 0.82)
Mortality	Control group	41,220	427	205,994	2.073	1	1	1	1
	DCIS group	13,740	198	68,505	2.890	1.40 (1.18, 1.65)	1.39 (1.18, 1.65)	1.42 (1.20, 1.68)	0.97 (0.80, 1.17)
	▪ Pure DCIS	6184	68	30,150	2.255	1.09 (0.85, 1.41)	1.07 (0.82, 1.38)	1.08 (0.84, 1.40)	0.94 (0.73, 1.22)
	▪ DCIS+Invasive	7556	130	38,355	3.389	1.63 (1.34, 1.98)	0.66 (1.36, 2.02)	1.70 (1.39, 2.07)	0.99 (0.78, 1.24)

*CI* confidence interval, *DCIS* ductal carcinoma in situ, *HR* hazard ratio

^a^Model 1 is crude

^b^Model 2 is adjusted for age at diagnosis

^c^Model 3 is adjusted for age at diagnosis, income, diabetes mellitus, hypertension, and dyslipidemia

^d^Model 4 is adjusted for age at diagnosis, income, residence, diabetes mellitus, hypertension, dyslipidemia, and Charlson’s comorbidity index score

When the DCIS group was divided into the pure DCIS and DCIS+Invasive groups, the trend of low CVD risk compared to the control group was sustained in each subgroup, even after adjusting for age, income, residence and chronic diseases (Table 2, Fig. 2). The pure DCIS group tended to have a lower MI risk and had a significantly lower stroke risk. While, patients in the DCIS+Invasive group had significantly lower incidence of both MI and stroke compared to the control group after multivariate adjustment (HR 0.42; 95% CI, 0.26–0.68 and HR 0.58; 95% CI, 0.41–0.82, respectively, model 4).. The mortality rate was similar to that of the control group in both pure DCIS and DCIS+Invasive groups (HR 0.94; 95% CI, 0.73–1.22 and HR 0.99; 95% CI, 0.78–1.24, respectively, model 4).

Age was categorized into two groups of < 50 and ≥ 50 years for the subgroup analysis of MI, stroke, and mortality risk in DCIS patients (Table 3). Among women with pure DCIS who were < 50 years at the DCIS diagnosis, the MI risk was similar to that of the control group (HR 0.91; 95% CI, 0.45–1.84), whereas women ≥50 years at diagnosis had a significantly lower MI risk (HR 0.51; 95% CI 0.27–0.95). In cases of the DCIS+Invasive group, women < 50 years at the DCIS diagnosis had a similar stroke risk to the control group (HR 0.79; 95% CI, 0.44–1.41), whereas women ≥50 years at the DCIS diagnosis had a non-significant trend for lower stroke risk (HR 0.67; 95% CI, 0.44–1.02). Women diagnosed of DCIS appeared to have similar mortality risk compared to the control group in all subgroups after multivariate adjustment (model 4).

**Table 3:** Risk of Myocardial Infarct, Stroke, and Mortality According to the Age of Diagnosis in the DCIS group

		Total *N*	Events (n)	Follow-up Duration (Person-Year)	Incidence rate per 1000	Model 1^a^ HR (95% CI)	Model 2^b^ HR (95% CI)	Model 3^c^ HR (95% CI)	Model 4^d^ HR (95% CI)
Myocardial Infarct	< 50 years old								
	Control group	25,560	67	130,119	0.515	1	1	1	1
	Pure DCIS	3837	9	18,966	0.475	0.92 (0.46, 1.85)	0.92 (0.46, 1.85)	0.97 (0.48, 1.95)	0.91 (0.45, 1.84)
	DCIS+Invasive	4683	5	24,281	0.206	0.40 (0.16, 0.99)	0.40 (0.16, 0.99)	0.41 (0.17, 1.03)	0.29 (0.11, 0.80)
	≥50 years old								
	Control group	15,660	126	75,381	1.671	1	1	1	1
	Pure DCIS	2347	11	11,137	0.988	0.59 (0.32, 1.10)	0.57 (0.31, 1.06)	0.56 (0.30, 1.03)	0.51 (0.27, 0.95)
	DCIS+Invasive	2873	16	14,025	1.141	0.68 (0.41, 1.15)	0.70 (0.42, 1.18)	0.68 (0.40, 1.14)	0.48 (0.27, 0.85)
Stroke	< 50 years old								
	Control group	25,560	97	130,071	0.746	1	1	1	1
	Pure DCIS	3837	8	18,961	0.422	0.57 (0.28, 1.18)	0.57 (0.28, 1.17)	0.59 (0.29, 1.22)	0.56 (0.27, 1.15)
	DCIS+Invasive	4683	20	24,228	0.825	1.10 (0.68, 1.78)	1.10 (0.68, 1.78)	1.13 (0.70, 1.84)	0.79 (0.44, 1.41)
	≥50 years old								
	Control group	15,660	212	75,174	2.820	1	1	1	1
	Pure DCIS	2347	24	11,114	2.159	0.77 (0.50, 1.17)	0.74 (0.49, 1.13)	0.72 (0.47, 1.10)	0.67 (0.44, 1.02)
	DCIS+Invasive	2873	27	14,010	1.927	0.68 (0.46, 1.02)	0.70 (0.47, 1.050	0.68 (0.46, 1.02)	0.49 (0.31, 0.75)
Mortality	< 50 years old								
	Control group	25,560	142	130,304	1.090	1	1	1	1
	Pure DCIS	3837	28	18,983	1.475	1.26 (0.91, 2.04)	1.36 (0.91, 2.04)	1.42 (0.94, 2.23)	1.21 (0.80, 1.83)
	DCIS+Invasive	4683	60	24,494	2.470	2.26 (1.67, 3.06)	2.26 (1.67, 3.06)	2.32 (1.72, 3.15)	1.16 (0.79, 1.69)
	≥50 years old								
	Control group	15,660	285	75,690	3.765	1	1	1	1
	Pure DCIS	2347	40	11,166	3.582	0.95 (0.69, 1.33)	0.91 (0.66, 1.27)	0.92 (0.66, 1.28)	0.81 (0.58, 1.13)
	DCIS+Invasive	2873	70	14,063	4.978	1.32 (1.02, 1.71)	1.37 (1.60, 1.78)	1.40 (1.08, 1.82)	0.85 (0.63, 1.15)

*CI* confidence interval, *DCIS* ductal carcinoma in situ, *HR* hazard ratio

^a^Model 1 is crude

^b^Model 2 is adjusted for age at diagnosis

^c^Model 3 is adjusted for age at diagnosis, income, diabetes mellitus, hypertension, and dyslipidemia

^d^Model 4 is adjusted for age at diagnosis, income, residence, diabetes mellitus, hypertension, dyslipidemia, and Charlson’s comorbidity index score

## Discussion

In this large population-based cohort study, we observed that women initially diagnosed with DCIS had higher comorbidity rates but fewer cardiovascular events (MI and stroke) compared to the matched control group. All-cause mortality did not differ between the DCIS group and control group after adjusting for age at diagnosis, income, residence and chronic diseases. The trend for CVD events differed according to subsequent invasive disease in DCIS patients < 50 years at diagnosis. The incidence of MI was similar in the pure DCIS group compared to the control group, whereas the DCIS+Invasive group had a lower MI risk. A tendency for a lower incidence of stroke in the pure DCIS patients was observed, whereas stroke risk was similar between the control group and the DCIS+Invasive group.

Although all-cause mortality of women initially diagnosed with DCIS was higher than the control group in the crude model, it was attenuated after adjustment for age at diagnosis, income, residence and comorbidities. This observation is consistent with previous population-based studies reporting similar or slightly lower mortality risk in pure DCIS patients compared to the general population [8, 9, 11, 12, 17, 18]. A notable finding in this study is that the increased mortality risk was attenuated after adjustment for comorbidities, even in women with a subsequent diagnosis of invasive disease (DCIS+Invasive group). This can imply that in early breast cancer, mortality risk is more influenced by comorbidities rather than invasive cancer, emphasizing the importance of comorbidity management.

In our study, women initially diagnosed with DCIS had a 49% significantly lower risk of MI compared to the control group. Data for cause-specific mortality was missing in our study, but the lower incidence can be presumed to lead to lower MI-related mortalities, similar to previous reports on cardiac-related mortality of DCIS patients in population-based studies [8, 9, 11, 12]. Whereas, in population-based studies that specifically assessed incidence of cardiac morbidities in DCIS patients, the risk was similar compared to the general population [11, 19]. The decreased risk of MI in DCIS patients in our study can be partly attributed to estrogens, as high levels of estrogens are a risk factor for DCIS but a protective factor for cardiovascular diseases [20].

The risk of MI in women < 50 years old with pure DCIS was similar to that of the control group, in comparison with the lower MI risk in other subgroups. It is difficult to compare this finding with those of previous studies in which the reported cardiovascular morbidity or mortality event rate was zero in women < 50 years old [8, 11]. A possible explanation is that women < 50 years old are less likely to be diagnosed with DCIS via a screening program, resulting in a lower rate of diagnosis and consequently poor management of comorbidities.

Similar to MI risk, the risk for stroke in women initially diagnosed with DCIS was 40% lower than the control group in our study. Previous studies have only reported mortality due to cerebrovascular diseases in DCIS patients, demonstrating lower risk compared to the general population [8, 11, 21]. Although, cause-specific mortality is unknown in our study, the lower stroke risk showed in our study is comparable to these reports. In contrary, studies including invasive breast cancers reported a small but increased risk of stroke in several population-based studies [22–26]. Whereas, in our study, patients with a subsequent diagnosis of invasive cancer demonstrated lower risk of stroke compared to the control group.

Although we did not find a lower all-cause mortality risk in DCIS patients compared to the control group in this study, we detected a lower CVD risk in DCIS patients, possibly reflecting a healthy user effect, which includes better access to health care, receiving better preventive care, and adopting a healthier lifestyle after the diagnosis [8, 27]. We found that DCIS patients were more socioeconomically advantaged and were more likely to live in an urban area, which would lead to better access to health care. Women diagnosed with DCIS are likely to show a ‘healthy adherer’ effect: a lower mortality risk in women who are more adherent to clinical trial drugs, whether the active drug or placebo [28, 29]. In our study, DCIS patients had higher rates of hypertension, DM, and dyslipidemia, probably as a result of surveillance bias. Most DCIS patients are diagnosed through screening programs, meaning that regular health checkups would have been done also. Frequent health checkups will result with higher rates of comorbidities, probably at an earlier stage. Paradoxically, this might explain the lower incidence of MI and stroke, as DCIS patients received treatment for these comorbidities, which will reduce their risk of CVDs. Also, good adherence to treatment and healthy behavior can also be expected after diagnosis of DCIS. Whereas a high proportion of the general population with comorbidities may not be diagnosed and thus remain untreated.

The strengths of our study include the large size, population-based character, and case-control cohort setting. Many population-based studies have compared disease incidence and risk with the general population. Our study designated a matched control group to compare comorbidities and socioeconomic status. This minimized the bias related to risk factors of mortality and cardiovascular diseases.

However, the Korean NHIS database is limited in providing treatment-related information. Data on adjuvant therapy were not included in our study. Exposing the heart to ionizing radiation is associated with an increased risk of subsequent cardiovascular disease [30, 31]. Although data on radiation therapy and its techniques were not included, this limitation was minimized, as modern radiation therapy techniques have been reported to maintain a low heart dose, not inducing significant excess risk for MI [11, 32]. Another weakness of our study is the lack of data regarding chemotherapy or endocrine therapy. In Korea, only tamoxifen is prescribed as adjuvant DCIS endocrine therapy. Previous studies have yielded conflicting findings about the relationship between tamoxifen use and MI risk, ranging from a protective effect to increased risk [33–35]. Tamoxifen increases the risk of venous thromboembolism, but the association with stroke risk is also conflicting in previous reports [36–38]. Certain chemotherapy regimens are known for their cardiotoxicity, and chemotherapy itself is a risk factor for stroke [39, 40]. However, our study cohort consisted of early-stage patients, who are likely to have a relatively low rate of chemotherapy administration, which minimized the limitation of the lack of these data.

Another weakness of this study is that cause-of-death data were missing, so we were unable to analyze the association among DCIS, CVD risk, and mortality. This data would be needed to analyze the reason for why the lower CVD risk in DCIS patients did not translate to lower mortality rate. In our study, MI and stroke events were only detected when a patient was hospitalized. MI or strokes that are too minor to require hospitalization, or events that are so severe that led to death before hospitalization, were not recorded. A relatively short follow-up duration of a median of 5.3 years is also a limitation. Previous studies reported that CVD risks differ according to follow-up time, and studies with longer follow-up durations are needed to verify the results of our study [9, 22, 24].

## Conclusions

In conclusion, women initially diagnosed with DCIS had a lower risk of MI and stroke compared to the control group, which may indicate differences in health behavior. A higher mortality risk in DCIS patients was attenuated after adjustment for age at diagnosis, income, residence and comorbidities. Healthcare providers should emphasize the importance of managing preexisting comorbidities along with DCIS treatment when counseling DCIS patients.

## Supplementary Information

**Additional file 1: Supplementary table.** Definitions of comorbidities and outcomes.

## Data Availability

The data that support the findings of this study are available from the National Health Insurance service (NHIS) but restrictions apply to the availability of these data, which were used under license for the current study, and so are not publicly available. Interested researchers can request access to the data from NHIS. The detailed information for data access of NHIS could be obtained from the NHIS website (www.nhis.or.kr, nhiss.nhis.or.kr).

## Abbreviations

CCI: Charlson Comorbidity Index
CI: Confidence interval
CVD: Cardiovascular disease
DCIS: Ductal carcinoma in situ
DM: Diabetes mellitus
HR: Hazard ratio
ICD-10: International Classification of Diseases, 10th Revision
MI: Myocardial infarct
NHIS: National Health Insurance System
SEER: Surveillance, Epidemiology, and End Results
SMR: Standardized mortality ratio
