# Supplementary material for: Cardiovascular events and mortality in a population-based cohort initially diagnosed with ductal carcinoma in situ
Source: BMC Cancer. 2021 Jun 26;21:735. doi: 10.1186/s12885-021-08494-0 (PMC8236151; doi:10.1186/s12885-021-08494-0)
Supplement: Supplementary file 1 — Additional file 1: Supplementary table. Definitions of comorbidities and outcomes. [file 12885_2021_8494_MOESM1_ESM.docx]

**Cardiovascular events and mortality in a population-based cohort initially diagnosed with ductal carcinoma in situ**

**Running title: Cardiovascular events and mortality in DCIS patients**

Tae-Kyung Yoo, MD^1,2^, SangHyun Park^3^, Kyung Do Han, PhD^4^, Byung Joo Chae, MD, PhD^5^*

^1^ Department of Surgery, Seoul St. Mary’s Hospital, College of Medicine, The Catholic University of Korea, Seoul, 06591, Republic of Korea

^2^ Cancer Research Institute, College of Medicine, The Catholic University of Korea, Seoul, 06591, Republic of Korea

^3^ Department of Medical Statistics, College of Medicine, The Catholic University of Korea, 06591, Republic of Korea

^4^ Department of Statistics and Actuarial Science, Soongsil University, Seoul 06978, Republic of Korea

^5^ Department of Surgery, Samsung Medical Center, Sungkyunkwan University, Seoul, 06351, Republic of Korea

Correspondence to:

Byung Joo Chae

Division of Breast Surgery, Department of Surgery, Samsung Medical Center, Sungkyunkwan University School of Medicine, 81 Irwon-Ro, Kangnam-Gu, Seoul 06531, Korea

Tel: +82-2-3410-3479, Fax: +82-2-3410-6982

Email: [bj.chae@samsung.com](mailto:bj.chae@samsung.com), [bjchae@gmail.com](mailto:bjchae@gmail.com)

**Supplementary table S1. Definitions of comorbidities and outcomes**

|  | **ICD-10 codes** | **Additional** **definitions** |
| --- | --- | --- |
| **Comorbidities** |  |  |
| • Hypertension | I10-I13, I15 | Claims for antihypertensive agents |
| • Diabetes mellitus | E11-E14 | Claims for oral antidiabetic agents (sulfonylureas, metformin, meglitinides, thiazolidinediones, dipeptidyl peptidase-4 inhibitors, Ɑ-glucosidase inhibitors) or insulin |
| • Dyslipidemia | E78 | Claims for agents for dyslipidemia |
| **Outcomes** |  |  |
| • Myocardial infarct | I21, I22 | Diagnosis during hospitalization ≥ 1 |
| • Stroke | I63, I64 | Diagnosis during hospitalization ≥ 1 with claims for brain imaging studies (brain CT or MRI) |
| **Charlson’s comorbidities index** | |  |
| • Congestive heart failure | I109.9, I11.0, I13.0, I13.2, I42.0, I42.5-I42.9, I43.x, I50.x, P29.0 |  |
| • Peripheral vascular disease | I70.x, I71.x, I73.1, I73.8, I73.9, I77.1, I79.0, I79.2, K55.1, K55.8, K55.9, Z95.8, Z95.9 |  |
| • Dementia | F00.x-F03.x, F05.1, G30.x, G31.1 |  |
| • Chronic pulmonary disease | I27.8, I27.9, J40.x-J47.x, J60.x-J67.x, J68.4, J70.1, J70.3 |  |
| • Rheumatologic disease | M05.x, M06.x, M31.5, M32.x-M34.x, M35.1, M35.3, M36.0 |  |
| • Peptic ulcer disease | K25.x-K28.x |  |
| • Mild Liver disease | B18.x, K70.0-K70.3, K70.9, K71.3-K71.5, K71.7, K73.x, K74.x, K76.0, K76.2-K76.4, K76.9, Z94.4 |  |
| • Diabetes without chronic complication | E10.0, E10.1, E10.6, E10.8, E10.9, E11.0, E11.1, E11.6, E11.8, E11.9, E12.0, E12.1, E12.6, E12.8, E12.9, E13.0, E13.1, E13.6, E13.8, E13.9, E14.0, E14.1, E14.6, E14.8, E14.9 |  |
| • Diabetes with chronic complication | E10.2-E10.5, E10.7, E11.2-E11.5, E11.7, E12.2-E12.5, E12.7, E13.2-E13.5, E13.7, E14.2-E14.5, E14.7 |  |
| • Hemiplegia or paraplegia | G04.1, G11.4, G80.1, G80.2, G81.x, G82.x, G83.0-G83.4, K83.9 |  |
| • Renal disease | I12.0, I13.1, N03.2-N03.7, N05.2-N05.7, N18.x, N19.x, N25.0, Z49.0-Z49.2, Z94.0, Z99.2 |  |
| • Any malignancy including leukemia and lymphoma | C00.x-C26.x, C30.x-C34.x, C37.x-C41.x, C43.x, C45.x-C58.x, C60.x-C76.x, C81.x-C85.x, C88.x, C90.x-C97.x |  |
| • Moderate or severe liver disease | I85.0, I85.9, I86.4, I98.2, K70.4, K71.1, K72.1, K72.9, K76.5, K76.6, K76.7 |  |
| • Metastatic solid tumor | C77.x-C80.x |  |
| • AIDS | B20.x-B22.x, B24.x |  |
